# Influence of Storage Packaging Type on the Microbiological and Sensory Quality of Free-Range Table Eggs

**DOI:** 10.3390/ani13121899

**Published:** 2023-06-06

**Authors:** Zofia Sokołowicz, Miroslava Kačániová, Magdalena Dykiel, Anna Augustyńska-Prejsnar, Jadwiga Topczewska

**Affiliations:** 1 Department of Animal Production and Poultry Products Evaluation, University of Rzeszów, Zelwerowicza Street 4, 35-601 Rzeszów, Poland; zsokolowicz@ur.edu.pl (Z.S.); aaugustynska@ur.edu.pl (A.A.-P.); 2 Institute of Horticulture, Faculty of Horticulture and Landscape Engineering, Slovak University of Agriculture, 949 76 Nitra, Slovakia; miroslava.kacaniova@gmail.com; 3 Department of Food Production and Safety, State University of Applied Sciences in Krosno, Rynek 1, 38-400 Krosno, Poland; magdalena.dykiel@pans.krosno.pl

**Keywords:** table eggs, storage, type of packaging, microbiological quality, sensory characteristics, functional properties of eggs

## Abstract

**Simple Summary:**

Eggs are a good source of high-quality protein, essential fatty acids, vitamins, and minerals. In addition to their high nutritional value, table eggs must be microbiologically safe and have favourable sensory and functional qualities. Factors that shape egg quality can be storage time and conditions. Marketable eggs may be packaged in plastic or cardboard egg packaging. The influence of the type of packaging, storage time, and temperature on the microbiological quality of the shell and contents of eggs and the foaming properties of the egg white as well as the sensory characteristics of the eggs after hard-boiling were investigated. The type of packaging was shown to influence the microbiological quality of the egg contents. More bacteria were found in the contents of eggs stored in plastic packaging than in cardboard packaging. The type of packaging in which the eggs were stored did not affect the foaming properties of the egg white or the sensory characteristics of the eggs after hard-boiling. Irrespective of the type of packaging, a tendency toward deterioration of the foaming properties of egg white and a deterioration of the sensory characteristics of eggs after hard-boiling was found with storage time. The storage of eggs at 5 °C was more favourable for their microbiological quality and the stability of egg white foam. Regardless of the type of packaging, eggs stored at 5 °C after hard-boiling had better yolk colour, smell, and texture than eggs stored at 22 °C.

**Abstract:**

The studies aimed to assess the impact of packaging, storage time, and temperature on the microbiological quality as well as on the sensory quality and functional properties of chicken eggs. The study material consisted of eggs from laying hens kept under free-range conditions. The eggs packed in cardboard and plastic cartons were stored at 5 °C and 22 °C, respectively. The eggs were examined on the day of laying and on days 14 and 28 of storage. The microbiological quality of the shell and contents of the eggs and the foaming properties of the egg white stored in cardboard and plastic packaging as well as the sensory characteristics of the eggs stored in both types of packaging after hard-boiling were examined on all evaluation dates. The type of packaging in which the eggs were stored was shown to influence the microbiological quality of the egg contents. Eggs stored in plastic packaging, on days 14 and 28 of storage, contained more bacteria in egg contents than eggs stored in cardboard packaging (*p* < 0.05). The type of packaging in which the eggs were stored did not have an effect on the foaming properties of the egg white (*p* > 0.05) or on the sensory characteristics of the eggs after hard-boiling. Irrespective of the type of packaging, the foaming properties of the egg white and the sensory characteristics of the eggs after hard-boiling deteriorated with storage time. The effect of temperature on egg quality was found. Regardless of the type of packaging, eggs stored at 5 °C after hard-boiling had better yolk colour, smell, and texture than eggs stored at 22 °C (*p* < 0.05).

## 1. Introduction

The wide availability, relatively low price, and high nutritional value of chicken eggs make them an integral part of human diet. Eggs are a good source of high-quality protein, the necessary unsaturated fatty acids and fat-soluble vitamins, vitamins of the B group, mineral compounds, and other biologically active compounds [1,2,3].

In addition to high nutritional value, food eggs should be microbiologically safe and have advantageous sensory and functional qualities. The production of good quality eggs is crucial to its profitability and consumer acceptance [4]. Today’s consumers are showing a strong interest in animal products including farming systems that take animal welfare into account [5], making the percentage of hens kept in alternative systems to cage farming increase significantly. With the increase in egg production in non-cage systems, there is concern about the increased risk of bacterial contamination of table eggs. Most of the eggs produced are consumed as fresh table eggs and are used as a major ingredient in the food industry, which can pose a serious risk to food safety [6,7,8]. Eggs from floor and free-range systems generally have more bacteria on the surface of the eggs than eggs from cage systems [9,10]. Furthermore, Tomczyk et al. [11,12] showed that the greatest diversity and abundance of microorganisms were found in eggshells laid by hens kept in barn and free-range systems.

Microbiological contamination can endanger the quality, shelf life, and safety of eggs [13]. The eggshell microflora can contain both microorganisms that cause the eggs to spoil and pathogenic microorganisms. Of the various potential sources of risk, egg consumption has been shown to account for a significant proportion of sporadic cases of salmonellosis [14,15]. The microbiological contamination of eggs can occur vertically or horizontally. Vertical contamination occurs when eggs are formed in the hen’s ovary or oviduct [16]. Horizontal contamination occurs when the bacteria penetrate through the eggshell after eggs are laid [17,18]. Although an egg has defensive mechanisms in the form of a cover with cuticle, shell membrane of the shell, and antimicrobial proteins in the albumen, and the albumen has high pH and viscosity that hinders the multiplication of bacteria [19], bacteria can still penetrate inside the eggs. Storage conditions and time are one of the main conditions determining the safety of food eggs. Changes that occur during storage can create favorable conditions for microbiological contamination of eggs. For example, the cuticle, which is an important protective barrier, in conditions of increased relative humidity of the eggshell surface, can be digested as a result of the glycolytic activity of some bacteria (e.g., *Pseudomonas*) [20]. Storage time and conditions bring about a number of physical and chemical changes in egg content which in turn affect the functional (technological) characteristics and antibacterial properties of egg albumen [21,22,23]. The changes occurring in the egg content are mainly a result of water migration between the yolk and the albumen and loss of water and carbon dioxide through the pores of the eggshell. As storage time gets longer, the albumen height is reduced; however, pH and foaming properties increase [24]. The strength of the vitelline membrane surrounding the yolk is also reduced [22], which in turn favors the movement of nutrients between the albumen and the yolk [25,26]. In addition, storage time and conditions cause the degradation of egg albumen [21,25,26] and, consequently, the reduction of its antibacterial properties [21]. Storage time and conditions can have a significant impact on the physicochemical characteristics, microbiological quality, and sensory characteristics of eggs. 

Consumers purchase raw eggs and egg products. Egg products account for around 20% of total egg consumption in Europe and range from 50% (149 eggs/person/year) in Denmark to 5% (eight eggs/person/year) in Poland [27]. Among egg products, hard-boiled eggs are the main item. 

Sensory qualities are an important attribute of the quality of hard-boiled eggs. Consumers expect the yolk of hardboiled eggs to be centred so that the shell can be removed without damaging the white, and the yolk is fully coagulated but without a greenish colour at the border between the cooked white and the yolk [28].

Sensory properties are a significant attribute of egg quality. Instrumental evaluation gives objective results in terms of individual egg quality parameters but does not allow us to predict how a consumer perceives the product. According to Meilgaard et al. [29], the sensory evaluation results allow measuring the consumer’s reaction to food; therefore, the sensory evaluation of eggs is important and even necessary. The sensory evaluation of eggs is a common assessment of their quality and acceptance by consumers [30]. Sensory evaluation most often takes into account color, taste, and odor [31]. Storage time and conditions can affect the sensory quality of eggs [32,33,34].

Chicken eggs have numerous functional properties that are used in the foaming of food production, and the albumen being one of the most important of them [35]. Chicken egg albumen is the most frequently used foaming agent in food technology [36,37,38,39,40,41]. The foam formation capacity and the stability of the foam during storage and thermal treatment are the main factors during the formulation of new recipes for aerated foods [42].

Storage time and conditions can affect the foam formation capacity and the foam stability. As egg storage time is extended, the foam stability is reduced; the foam whipped from fresh eggs is more stable than the foam whipped from eggs after storage time [43]. 

Eggs in trade and with consumers are most often stored in plastic or cardboard packages (10 pieces each). 

The studies aimed to assess the influence of the type of packaging, storage time, and temperature on the microbiological quality of the shell and contents of eggs and the foaming properties of the egg white as well as the sensory characteristics of the eggs after hard-boiling were investigated. 

## 2. Materials and Methods

### 2.1. Egg Samples 

The study material consisted of 450 eggs (microbiological tests of the shell and egg contents 90, sensory evaluation of hard-boiled eggs 135, and egg white foam quality 225) from laying hens kept in barn conditions with free-range system. The eggs were purchased on the day of laying at one of the poultry farms. 50 eggs were evaluated on the day of laying, and the remaining 400 eggs were packed into 20 cardboard extruders of 10 eggs each and 20 plastic extruders of 10 eggs each. Eggs packed in cardboard and plastic cartons were stored: 10 cartons × 10 eggs = 100 at 5 °C (refrigerated cabinet FKv36/10, Liebherr, Ulm, Germany) and 10 cartons × 10 eggs = 100 at 22 °C (on a countertop under room conditions) and evaluated on the 14th and 28th day of storage. The microbiological quality of the shell and contents of the eggs, the sensory characteristics of the eggs after hard boiling, and the technological properties of the egg white were examined on all evaluation dates.

### 2.2. Microbiological Analysis

Using a swab, the surface bacterial count was measured. The entire egg’s surface was aseptically swabbed with a sterile cotton swab before being diluted with ordinary saline. The samples were serially further diluted, and 100 µL of each dilution was applied on the plate count agar (PCA) surface (Oxoid, Basingstoke, UK). Samples were dipped in 75% ethanol for 5 min and then left to air dry before being used to count the number of bacteria in egg contents. The egg’s upper end was exposed to flame for 5–10 s before being punctured using a sterile tool. After serial dilution for an aerobic bacterial count, the entire egg’s contents were combined in a sterile polythene bag and then placed on a PCA. The Petri dishes with the inoculum were incubated at 30 °C for 48–72 h. After serial dilution, the surface and contents (100 µL) were transferred to lactose-free violet red bile agar (VRBL, Oxoid, Basingstoke, UK) and incubated for 24–48 h at 37 °C.

Before detection, the microbial colonies were subcultured on TSA agar (Tryptone Soya Agar, Oxoid, UK) for 18 to 24 h at 37 °C. A colony was formed from eight different bacterial strains. According to Kačániová et al. [44], the identification was then performed using the Maldi TOF-MS Biotyper. In total, 217 isolates were found to have a value higher than 2.

### 2.3. Sensory Evaluation

The evaluation of the sensory characteristics of hard-boiled eggs was carried out in the laboratory of Poultry Products Evaluation. Evaluation was carried out on the day of laying and after 14 and 28 days of storage. Fifteen participants participated in the evaluation (six students aged 22 to 23 years and nine university employees aged 27 to 56 years). Before the first evaluation date, the panellists underwent a three-hour training session during which they assessed the sensory characteristics of the eggs: overall appearance, yolk colour, smell, taste, and texture. The study used specially prepared evaluation sheets to describe the sensory properties of the samples. All eggs for organoleptic evaluation were prepared the same way. Two hours prior to the start of the evaluation, the eggs were hard boiled. To do this, the eggs were placed in water at room temperature, brought to a boil, and boiled for 15 min, then removed from the pot and placed under cold running water for 10 min. After being cooled in cold water, the eggs were peeled from their shells, cut lengthwise into 2 halves, and placed in separate containers labelled with a letter-number code. Containers were covered until the assessment began. The test was conducted in a suitably prepared room free of foreign odours at 22 °C. The evaluation of each sample was carried out on a 9-point linear hedonic scale where 1 point represented the worst evaluation, 5 the indifferent evaluation, and 9 the best evaluation. Before the assessment, panellists were asked not to eat for three hours prior to the test. Panellists received mineral water and salt-free crackers to scrub their tongues and gums between sample tests.

### 2.4. Technological Properties of Egg White

The foaming properties were evaluated using foaming capacity (FC) and foam stability (FS) based on the method described by Ferreira et al. [45] with some modifications.

The foams were processed by whipping 100 mL of albumen for 3 min at 20 °C with a mixer operating at 1220 rpm. The volumes of foam and the drained liquid were evaluated just after whipping and during holding for 30 min at 20 °C. For the determination of FC and FS, the following formulae are used:

FC (%) = (FV/ILV) × 100%

FS (%) = [(ILV − DV)/ILV] × 100%

where: FV—volume of foam; ILV—volume of the initial; liquid phase; DV—volume of drainage.

### 2.5. Statistical Analysis

All statistical analyses were performed using Statistica 13.3 [46]. The effects of packaging type, temperature, and storage time on the microbiological quality and sensory characteristics of eggs and technological characteristics of egg white were verified using ANOVA analysis. Significant differences between mean trait values were estimated using the Duncan test. Results were considered statistically significant at *p* < 0.05. The results obtained were subjected to a multivariate analysis of variance. The main effect was determined: the influence of P, packaging type, T, storage temperature and S–storage time, and the interaction between factors (P×T, P×S, T×S, P×T×S). 

## 3. Results and Discussion

In cardboard box, the total count of bacteria (TCB) on eggshell ranged from 1.43 to 2.63 log cfu/mL; in plastic box, the total count of bacteria (TCB) ranged from 1.75 to 2.76 log cfu/mL (Table 1). In cardboard box, the total count of bacteria (TCB) in internal egg content ranged from 0.00 to 1.92 log cfu/mL; in plastic box, total count of bacteria (TCB) in internal egg content ranged from 0.00 to 2.49 log cfu/mL (Table 1). In cardboard box, the number of coliform bacteria (CB) on eggshell ranged from 0.00 to 2.15 log cfu/mL and in plastic box from 0.00 to 1.94 log cfu/mL. In cardboard box, the number of coliform bacteria (CB) in internal egg content ranged from 0.00 to 1.37 log cfu/mL; in plastic box, the number of coliform bacteria (CB) ranged from 0.00 to 1.80 log cfu/mL (Table 1). The study found an effect of packaging type on the total count of bacteria (TCB) and coliform bacteria (CB) in internal egg content (*p* < 0.05) (Table 1.). There was no effect of packaging type on the total count of bacteria (TCB) and coliform bacteria (CB) on eggshell (*p* > 0.05).

Factors influencing the quality of eggs after laying are mainly egg storage time and temperature [43] but also the kind of packaging [47]. The study found an effect of storage time on the total count of TCB) and coliform bacteria (CB) both on the shell surface and in the egg contents (*p* > 0.05). At all assessment dates, bacterial counts were higher at 22 °C than at 5 °C (Table 1). The results obtained are in agreement with the findings of Chousalkar et al. [48] that showed an increase in the ambient temperature resulted in an increase in the bacterial replication rate. The total count of bacteria on the surface of the shell and in the contents of eggs stored in both types of packaging did not exceed acceptable levels, and non-pathogenic bacteria predominated among the bacteria isolated. In this study, 217 isolates were identified from 31 species, 11 genera, and 10 families. The most isolated species was *Herbaspirillum huttiense* (42 isolates) (Table 2).

**Figure 1:**
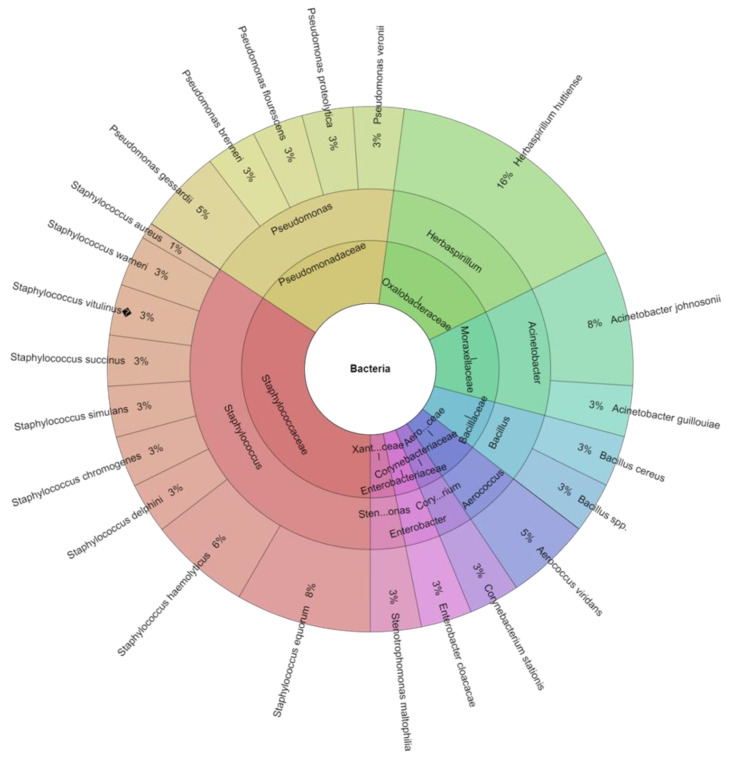
Percentage of isolated species of bacteria in cardboard box.

**Figure 2:**
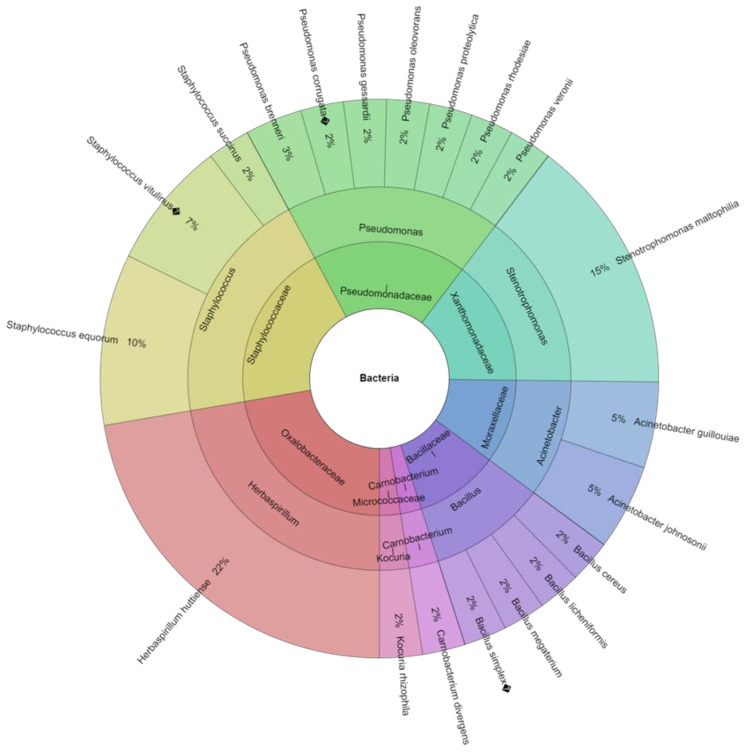
Percentage of isolated species of bacteria in plastic box.

Among the bacteria identified, the most dangerous bacteria for human health, such as *Salmonella* spp., *Campylobacter* spp., and *Listeria* spp., were not found. The majority of the bacteria found on the eggshell are Gram-positive. Gram-negative bacteria are better adapted to invading the barriers of the eggshell and membranes, leading to spoiled eggs [49], but a few Gram-positive bacteria can also be present. The most common contaminants come from the genera *Pseudomonas*, *Alcaligenes*, *Proteus*, *Arthrobacter*, *Escherichia*, *Serratia*, *Aeromonas*, *Hafnia*, *Citrobacter*, *Salmonella*, *Micrococcus*, *Staphylococcus*, and *Bacillus*. The most important bacteria in foodborne diseases transmitted to humans from chicken eggs are from *Salmonella* spp., *Campylobacter* spp., *Listeria* spp., *Staphylococcus* spp., and *Escherichia* spp. Fecal material, soil, and dust are the principal sources of contamination to which eggs are exposed [50]. Eggs can become contaminated by microorganisms at various points along the manufacturing, processing, preparation, and consumption chains. When eggs become infected while they are developing in the hen’s ovaries, this is known as transovarian or "vertical" transfer of microorganisms. When microorganisms penetrate the eggshell and are then subjected to a contaminated environment, horizontal transmission takes place. Others disagree with Barrow and Lovell’s [51] studies that claim the most contamination is caused by horizontal spread [52]. In cardboard boxes containing eggs, 23 species of bacteria from 9 genera and 9 families were discovered (Figure 1). The most isolated species was *Herbaspirillum huttiense* (16%). From plastic box stored eggs were isolated 20 species of bacteria, from 8 genera and 8 families (Figure 2). The most isolated species was again *Herbaspirillum huttiense* (22%). *Herbaspirillum huttiense* bacteria belong to the genus *Herbaspirillum* and are widely distributed in the environment [53]. They were first described by Baldani et al. [54] as well as Obradovic et al. [55] and Dobrista et al. [56]. Although *Herbaspirillum* bacteria are widely distributed in the environment, they are very rarely associated with infections in humans [57]. *Herbaspirillum* species, which occupy the roots of plants in the rhizosphere and have been discovered in wells and other ground water, are nitrogen-fixing bacteria [58,59,60]. They convert atmospheric nitrogen (N_2_) into ammonium ions (NH_4_^+^), a form that can be taken up by plants. These bacteria are found in the roots of economically important crops, such as wheat and maize, stimulating their growth by fixing nitrogen [61]. Among the microbiota colonising the shells of the eggs tested, *Staphylococcus aureus* bacteria were also found, and in the study [62], it is the main microorganism causing egg spoilage [63]. The presence of *Staphylococcus aureus* on eggshells increases the likelihood of this pathogen entering the egg contents, leading to an increase in risk of foodborne illness. Eggshell contamination is associated with the presence of bird droppings and feathers in poultry farms [64,65]. *Stenotrophomonas maltophilia* was also found on the surface of the shell and in the contents of the eggs. These microorganisms can cause food spoilage and infectious diseases in consumers once they enter the food chain [66,67,68,69]. In a study conducted, the number of *Stenotrophomonas maltophilia* bacteria was higher in eggs stored in plastic packaging than in cardboard packaging. The higher number of *Stenotrophomonas maltophilia* bacteria can be probably explained by their propensity to adhere to plastics and form biofilms [70].

Hard-boiled eggs are valuable ready-to-use egg products [2]. In various countries around the world, they are used for direct consumption or used as ingredients in other food products [71]. Consumers of hard-boiled table eggs are most interested in their taste and appearance [72,73]. The study showed no effect of the type of packaging during storage on the sensory characteristics of the eggs (*p* > 0.05) after hard-boiling. The evaluators gave similar scores for the individual characteristics of eggs (overall appearance, yolk colour, smell, taste, and texture) stored in cardboard and plastic packaging (Table 3).

Our study showed an effect of storage time on all the sensory characteristics of the eggs studied (*p* < 0.05) (Table 3). On day 14 of storage, the scores given by the panellists for the overall appearance, smell, taste, and texture of the eggs, both from cardboard and plastic packaging, at 5 °C were higher than on the first evaluation date (*p* < 0.05). On the 28th day of storage, the overall appearance, smell, taste, and texture of the eggs were rated lower than on earlier evaluation dates. Furthermore, the results of Guo et al. [32] on sensory evaluation showed that the eggs had satisfactory sensory evaluation results on the first day after laying and deteriorated with increasing storage time. The results of Attia et al. [34], who studied the sensory characteristics of eggs, showed that fresh eggs were superior in quality to eggs stored for 21 days. The effect of temperature on the sensory characteristics of eggs was found in our own research. In both cardboard and plastic packaging, the marks awarded for yolk colour, smell, and texture of eggs were higher for eggs stored at 5 °C than at 22 °C (*p* < 0.05). The poorer sensory evaluation results of eggs stored at room temperature (22 °C) can be linked to the faster rate of physico-chemical transformation of the egg contents during storage. Moreover, the results of the evaluation of the sensory characteristics of the eggs carried out by Attia et al. [34] showed that eggs stored at 5 °C were better than eggs stored at 23 °C. According to Alshaikhi et al. [33], poorer results in the evaluation of the sensory characteristics of eggs during storage at higher temperatures are due to an increase in evaporation and migration of water from the white to the yolk, leading to a reduction in the concentration of nutrients in the yolk. In a study by Aguinaga Bósquez et al. [74], based on human sensory evaluation, there was no effect of storage time on the characteristics of cooked eggs; however, the use of advanced analytical methods, such as e-tongue and e-nose, showed an effect of storage time (0, 30, and 60 days) on the sensory characteristics of eggs.

There was no effect of packaging type on the foaming capacity and foaming stability of egg white foam (Table 4). The foaminess and stability of foam from eggs stored in plastic and cardboard packaging was similar (*p* > 0.05) at all assessment dates. The foaminess of egg white foam at 5 and 22 °C was similar (*p* > 0.05). There was an effect of storage temperature on foam stability, with better stability of egg white foam stored at 5 °C than at 22 °C (*p* < 0.05). There was an effect of storage time on egg white foaminess and foam stability (*p* < 0.05). After 28 days of storage, the foaminess of the egg white and the stability of the foam was lower than at the first evaluation date. Foam stability was found to decrease with increasing storage time. The deterioration in foam stability can be explained by the bubble disproportioning processes taking place [35,75,76]. Drainage is also a phenomenon that weakens the foam structure. Water running off the surface of bubbles removes proteins from the interfacial boundaries, and the film becomes too thin to hold the bubble.

## 4. Conclusions

The results obtained indicate that the type of packaging in which the eggs are stored does not affect the foaming properties of the egg white and their sensory characteristics after hard-boiling but can affect the microbiological quality of the eggs. More bacteria were found in the contents of eggs stored in plastic packaging than in cardboard packaging. Irrespective of the type of packaging, a tendency for deterioration of the foaming properties of egg white and deterioration of the sensory characteristics of eggs after hard-boiling was found with storage time. 

The results obtained may be of relevance for optimising the selection of commercial egg packaging. However, further research is required for a full assessment of the impact of packaging type on the quality of stored eggs.

## Figures and Tables

**Table 1:** Influence of packaging type on the microbiological quality of eggs stored in different thermal conditions.

Storage Times (Days)	Kind of Package				*p*-Value for Main Effects	
	Cardboard Box		Plastic Box			
	5 °C	22 °C	5 °C	22 °C		
TCB Egg shell (log cfu/mL)					P T S P×T P×S T×S P×T×S	0.53 0.09 0.000 0.041 0.79 0.25 0.040
0	^x^ 1.78	^x^ 1.80	^x^ 1.75	^x^ 1.76		
14	^x^ 1.43 ^a^	^x^ 1.86 ^a^	^x^ 1.82 ^a^	^x^ 2.13 ^b^		
28	^y^ 2.55	^y^ 2.63	^y^ 2.40 ^a^	^y^ 2.76 ^b^		
CB Internal egg content (log cfu/mL)					P T S P×T P×S T×S P×T×S	0.010 0.26 0.005 0.10 0.16 0.53 0.45
0	^x^ 0.00	^x^ 0.00	^x^ 0.00	^x^ 0.00		
14	^y^ 1.59 ^a^	^y^ 1.92 ^a^	^y^ 2.11	^y^ 2.35 ^b^		
28	^y^ 1.60	1.73	^y^ 2.04	^y^ 2.49		
CB Egg shell (log cfu/mL)					P T S P×T P×S T×S P×T×S	0.17 0.51 0.000 0.000 0.21 0.22 0.000
0	^x^ 0.00	^x^ 0.00	^x^ 0.00	^x^ 0.00		
14	0.52	^x^ 1.12	^x^ 0.52	^x^ 1.00		
28	^y^ 1.37 ^ac^	^y^ 2.15 ^b^	^y^ 1.22 ^ac^	^y^ 1.94 ^bc^		
CB Internal egg content (log cfu/mL)					P T S P×T P×S T×S P×T×S	0.014 0.037 0.018 0.33 0.09 0.13 0.68
0	0.00	^x^ 0.00	0.00	^x^ 0.00		
14	0.00 ^a^	^y^ 1.37 ^a^	0.00 ^a^	^y^ 1.78 ^ac^		
28	0.52 ^ac^	^y^ 1.00 ^a^	1.64 ^c^	^y^ 1.80 ^b^		

Explanation: TCB—total number of bacteria, CB—coliform bacteria; P—effect of kind of package, T—effect of temperature, S—effect of storage duration; x, y values in columns with different letters differ significantly; a, b, c values in rows with different letters differ significantly (*p* < 0.05).

**Table 2:** Number of isolated species of bacteria.

Bacterial Species	Cardboard Box			Plastic Box			Total
	0 Day	14th Day	28th Day	0 Day	14th Day	28th Day	
*Acinetobacter guillouiae*		3		3	3		9
*Acinetobacter johnosonii*		5	3		3	3	14
*Aerococcus viridans*	5						5
*Bacillus cereus*	3			3			6
*Bacillus licheniformis*					3		3
*Bacillus megaterium*				3			3
*Bacillus simplex*				3			3
*Bacillus* spp.	3						3
*Carnobacterium divergens*					3		3
*Corynebacterium stationis*	3						3
*Enterobacter cloacacae*		3					3
*Herbaspirillum huttiense*		5	10	2	10	15	42
*Kocuria rhizophila*						3	3
*Pseudomonas brenneri*		3			4		7
*Pseudomonas corrugata*					3		3
*Pseudomonas flourescens*		3					3
*Pseudomonas gessardii*		5			3		8
*Pseudomonas oleovorans*					3		3
*Pseudomonas proteolytica*		3			3		6
*Pseudomonas rhodesiae*					3		3
*Pseudomonas veronii*		3			3		6
*Staphylococcus aureus*	1						1
*Staphylococcus delphini*	3						3
*Staphylococcus equorum*	5		3	2	5	5	20
*Staphylococcus haemolyticus*	3	3					6
*Staphylococcus chromogenes*		3					3
*Staphylococcus simulans*	3						3
*Staphylococcus succinus*	3			3			6
*Staphylococcus vitulinus*	3			3	3	3	12
*Staphylococcus warneri*	3						3
*Stenotrophomonas maltophilia*			3	3	10	5	21
Total	38	39	19	25	62	34	217

**Table 3:** Influence of packaging type on the sensory characteristics of eggs.

Trait	Time	Kind of Package				*p*-Value for Main Effects	
		Cardboard Box		Plastic Box			
		5 °C	22 °C	5 °C	22 °C		
overall appearance	1	^x^ 7.93 ± 0.96	^x^ 7.93 ± 0.96	^x^ 7.93 ± 0.96	^x^ 7.93 ± 0.96	P T S P×T P×S T×S P×T×S	0.21 0.06 0.000 0.51 0.61 0.39 0.81
	14	^x^ 8.27 ± 0.79 ^a^	^x^ 8.00 ± 0.84	^x^ 8.13 ± 1.06	7.40 ± 1.12 ^b^		
	28	^y^ 7.20 ± 1.08	^y^ 6.93 ± 1.16	^y^ 7.06 ± 1.17	^y^ 6.67 ± 0.98		
	1–28	7.80 ± 1.03	7.62 ± 1.09	7.71 ± 1.14	7.33 ± 1.13		
yolk colour	1	8.13 ± 0.99	^x^ 8.13 ± 0.99	^x^ 8.13 ± 0.99	^x^ 8.13 ± 0.99	P T S P×T P×S T×S P×T×S	0.49 0.007 0.000 1.00 0.87 0.16 0.41
	14	8.07 ± 0.88	^x^ 7.67 ± 1.05	^x^ 8.20 ± 0.86 ^a^	7.27 ± 1.39 ^b^		
	28	7.67 ± 0.89	^y^ 6.73 ± 1.16	^y^ 7.20 ± 1.08	^y^ 6.80 ± 1.61		
	1–28	7.96 ± 0.93 ^a^	7.51 ± 1.19	7.84 ± 1.06	7.40 ± 1.44 ^b^		
smell	1	8.13 ± 0.99	^x^ 8.13 ± 0.99	8.13 ± 0.99	^x^ 8.13 ± 0.99	P T S P×T P×S T×S P×T×S	0.93 0.000 0.000 0.59 0.69 0.017 0.98
	14	^x^ 8.53 ± 0.63 ^a^	^x^ 7.80 ± 1.15 ^bc^	^x^ 8.53 ± 0.74 ^ab^	7.47 ± 1.19 ^c^		
	28	^y^ 7.60 ± 1.05 ^a^	^y^ 6.80 ± 1.16 ^b^	^y^ 7.80 ± 0.56 ^a^	^y^ 6.87 ± 0.99 ^b^		
	1–28	8.09 ± 0.97 ^a^	7.58 ± 1.22 ^b^	8.16 ± 0.82 ^a^	7.49 ± 1.16 ^b^		
taste	1	7.80 ± 1.21	^x^ 7.80 ± 1.21	7.80 ± 1.21	7.80 ± 1.21	P T S P×T P×S T×S P×T×S	0.85 0.05 0.005 0.67 0.96 0.40 0.93
	14	8.00 ± 1.25	7.67 ± 0.89	8.07 ± 0.16	7.40 ± 1.40		
	28	7.40 ± 1.55	^y^ 6.93 ± 1.16	7.47 ± 1.12	6.87 ± 1.00		
	1–28	7.73 ± 1.34	7.47 ± 1.14	7.78 ± 1.16	7.36 ± 1.25		
texture	1	^x^ 7.80 ± 1.20	^x^ 7.80 ± 1.20	^x^ 7.80 ± 1.20	^x^ 7.80 ± 1.20	P T S P×T P×S T×S P×T×S	0.09 0.002 0.000 0.67 0.39 0.039 0.47
	14	^y^ 8.74 ± 0.60 ^a^	^x^ 7.87 ± 0.99 ^b^	^x^ 8.33 ± 0.97 ^ab^	7.20 ± 1.02 ^c^		
	28	^x^ 7.40 ± 1.12	^y^ 6.60 ± 1.12	^y^ 6.80 ± 1.15	^y^ 6.67 ± 0.82		
	1–28	7.98 ± 0.14 ^a^	7.42 ± 1.23 ^b^	7.64 ± 0.26	7.22 ± 1.10 ^b^		

Explanation: P—effect of kind of package, T—effect of temperature, S—effect of storage duration; x, y, values in columns with different letters differ significantly; a, b, c values in rows with different letters differ significantly (*p* < 0.05).

**Table 4:** Influence of packaging type on foaming properties of egg white.

Trait	Time	Kind of Package				*p*-Value for Main Effects	
		Cardboard Box		Plastic Box			
		5 °C	22 °C	5 °C	22 °C		
Foaming capacity (%)	1	^x^ 516.00 ± 36.27	^x^ 516.00 ± 36.27	^x^ 516.00 ± 36.27	^x^ 516.00 ± 36.27	P T S P×T P×S T×S P×T×S	0.36 0.06 0.000 0.34 0.76 0.035 0.78
	14	^x^ 523.00 ± 35.06 ^a^	497.50 ± 30.39	^x^ 519.50 ± 24.55 ^a^	^y^ 480.50 ± 25.76 ^b^		
	28	^y^ 470.50 ± 25.32	^y^ 480.00 ± 37.19	^y^ 474.50 ± 23.15	^y^ 465.50 ± 27.43		
	1–28	503.17 ± 39.36	497.83 ± 36.71	503.33 ± 34.55	487.00 ± 36.43		
Foam stability (%)	1	^x^ 95.41 ± 2.29	^x^ 95.41 ± 2.29	^x^ 95.41 ± 2.29	^x^ 95.41 ± 2.29	P T S P×T P×S T×S P×T×S	0.56 0.039 0.000 0.42 0.15 0.32 0.56
	14	^x^ 93.88 ± 3.05 ^a^	^y^ 92.57 ± 2.34	^y^ 92.29 ± 2.05	^y^ 91.04 ± 2.35 ^b^		
	28	^y^ 90.79 ± 3.36	^z^ 87.91 ± 3.57	^y^ 90.38 ± 3.24	^y^ 89.76 ± 2.13		
	1–28	93.36 ± 3.44	91.96 ± 4.15	92.69 ± 3.26	92.07 ± 3.28		

Explanation: P—effect of kind of package, T—effect of temperature, S—effect of storage duration; x, y, z values in columns with different letters differ significantly; a, b values in rows with different letters differ significantly (*p* < 0.05).

## Data Availability

The data that support the findings of this study are available from the corresponding author (J.T.) upon reasonable request.
